# Uncoupling Protein 1 Promotes Nile Tilapia Resistance to Acute Cold Stress by Regulating Liver Metabolism

**DOI:** 10.3390/metabo15100668

**Published:** 2025-10-13

**Authors:** Meiqing Li, Jirong Jia, Chenguang Liu, Ran Cai, Yang Yu, Xiaozheng Yu, Wei Feng, Caiyun Sun, Wensheng Li

**Affiliations:** State Key Laboratory of Biocontrol, Guangdong Province Key Laboratory for Aquatic Economic Animals, South China Sea Bio-Resource Exploitation and Collaborative Innovation Center, School of Life Sciences, Sun Yat-Sen University, Guangzhou 510275, China; limq53@mail2.sysu.edu.cn (M.L.); jiafan2796@126.com (J.J.); chgliu1413@163.com (C.L.); alan6382@163.com (R.C.); yuyang65@mail2.sysu.edu.cn (Y.Y.); 15058173090@163.com (X.Y.); fengw23@mail2.sysu.edu.cn (W.F.); suncaiy@mail.sysu.edu.cn (C.S.)

**Keywords:** uncoupling protein 1, cold stress, Nile tilapia, siRNA, BAM15

## Abstract

**Background**: Low temperature stress is a major environmental challenge affecting the growth, metabolism, and survival of many aquaculture species, including Nile tilapia (*Oreochromis niloticus*). Understanding the molecular mechanisms underlying cold tolerance is therefore essential for improving fish resilience and aquaculture sustainability. **Methods**: In the present study, an acute cold stress model of Nile tilapia (*Oreochromis niloticus*) was established and it was found that uncoupling protein 1 (UCP1) was involved in the acute cold stress process of tilapia. **Results**: The upregulation of UCP1 in the liver under cold stimulation was regulated by stress hormones such as cortisol and adrenaline. UCP1 has a short half-life and is degraded by proteasomes. In tilapia primary hepatocytes, the addition of adrenergic receptor agonists resulted in mitochondrial membrane potential decreasing, while UCP1 siRNA transfection inhibited mitochondrial membrane potential. Biochemical characteristics indicate that UCP1 is a channel protein that mediates proton leakage. In addition, feeding and intraperitoneal injection of mitochondrial uncoupling agent BAM15 can alleviate the low-temperature stress of tilapia. **Conclusions**: UCP1 helps maintain the metabolic homeostasis of tilapia under acute cold stimulation and provides new insights into the mechanisms of cold resistance as well as potential treatment strategies in fish.

## 1. Introduction

Water temperature is one of the most critical environmental factors influencing the physiology, metabolism, and behavior of aquatic organisms. Exposure to low temperature disrupts normal metabolic processes [1], weakens immune function [2], induces oxidative stress and cellular damage [3], and ultimately reduces the survival rate of fish. Nile tilapia (*Oreochromis niloticus*), characterized by its rapid growth and strong resistance to environmental stress, is one of the most commercially important freshwater fish worldwide [4]. The most suitable water temperature for tilapia growth is 25–35 °C, and the lethal temperature is about 10 °C [5]. Consequently, massive losses often occur during winter, making the improvement of cold tolerance a key challenge for the sustainable development of tilapia aquaculture.

With the advancement of high-throughput sequencing and proteomics technologies, new insights have been gained into gene expression changes under low-temperature stress [5,6]. Cold stress triggers complex physiological and biological processes, including mitochondrial functionality, metabolism of lipids and carbohydrates, antioxidant mechanisms, programmed cell death (apoptosis), and protein degradation [7]. Although numerous studies have explored these responses in tilapia [8,9], the key transcription factors and early-response genes regulating cold tolerance remain largely unknown. Therefore, more fundamental studies are required to elucidate the molecular mechanisms underlying cold adaptation in fish.

Mitochondria play a central role in cellular energy metabolism and homeostasis. Mitochondrial dysfunction can lead to insufficient ATP production, excessive reactive oxygen species, impaired immune function, and reduced survival under stress conditions [10,11]. Uncoupling proteins (UCPs) are mitochondrial inner membrane proteins that can dissipate the proton gradient, thereby regulating energy metabolism, reducing oxidative stress, and contributing to thermal adaptation. In mammals, the UCP family comprises UCP1–UCP6, among which UCP1 is a key thermogenic protein that maintains body temperature during cold exposure [12]. In addition to mammals, the UCPs have been found in both fish and plants [13]. Unlike mammals where UCP1 is mainly expressed in brown adipose tissue, in fish, UCP1 expression is highest in the liver [14]. The regulation of this protein varies with environmental and physiological factors such as dietary restriction and cold exposure, and the extent of regulation differs among tissues and species. Illustratively, in the liver of the Common carp (*Cyprinus carpio*), UCP1 mRNA peaked during summer but declined in winter [15]. However, ucp1 and ucp2 in the liver, ucp3 in the muscle of Chinese perch (*Siniperca chuatsi*) were significantly upregulated after long-term cold exposure [14]. Moreover, UCPs have been shown to maintain cellular homeostasis in the brain of zebrafish under cold stimulation [16].

To date, most research on UCP1 in fish has focused on molecular cloning, tissue-specific expression, and expression changes under environmental challenges, whereas functional studies remain scarce [14,16,17,18]. Therefore, the purpose of this study was to investigate whether UCP1 participates in cold response and whether mitochondrial uncoupling to enhancing cold resistance by regulating liver metabolism of Nile tilapia.

## 2. Materials and Methods

### 2.1. Animals

All fishes were obtained from Guangdong Tilapia Breeding Farm (Guangdong Province, China). Nile tilapia were reared in recirculating freshwater tanks at 20 fishes per tank (40 cm × 50 cm × 60 cm), acclimated for ≥1 week at 28 °C under natural light cycles, and fed a commercial diet twice daily to satiation. Animal housing, use, and experimentation were approved by the Institutional Animal Care and Use Committee of Sun Yat-Sen University (Approval No: SYSU-IACUC-2023-B0453).

### 2.2. Molecular Identification of UCP1 in Nile Tilapia

The predicted nucleotide sequence of Nile tilapia UCP1 was obtained from the NCBI database (https://www.ncbi.nlm.nih.gov). RNA extraction and complementary DNA generation were performed as described previously [19]. Briefly, total RNA was isolated from different tissues using Trizol reagent (Invitrogen, Carlsbad, CA, USA). The NANODROP 2000 Spectrophotometer was used to determine the quantity of total RNA (Thermo, Waltham, MA, USA). The cDNAs were synthesized using M-MLV Reverse Transcriptase (Invitrogen, USA). The primers used for the UCP1 ORF clone were synthesized based on the predicted nucleotide sequence of the UCP1 (XM_005456938) and are shown in Table 1. PCR was used for the UCP1 clone. PCR products were purified with the E.Z.N.A. Gel Kit (OMEGA Bio-tek, Norcross, GA, USA) and ligated to the pCR2.1 vector for sequencing.

### 2.3. Tissue Distribution of Nile Tilapia UCP1

Tissue (telencephalon, mesocerebrum, metencephalon, medulla oblongata, hypothalamus, pituitary, head kidney, kidney, liver, spleen, foregut, midgut, hindgut, fat, red muscle, white muscle) samples were collected in RNase-free Eppendorf, frozen by liquid nitrogen, and stored at −80 °C before extracting RNA. Semi-quantitative RT-PCR was performed for UCP1 mRNA. For the PCR procedure, 35 cycles of amplification were used for UCP1, and 20 cycles for 18S rRNA.

### 2.4. Cold Stress Treatment of Nile Tilapia

To investigate the specific impact of acute cold stress on function of the UCP1, Nile tilapia previously acclimated to 28 °C were subjected to a rapid temperature decline from 28 °C to 13 °C over a period of one hour with ice; the temperature was maintained at this level for 24 h using a Rising CL650 aquarium chiller (Rising, Shenzhen, China) [20]. The experimental temperature is determined based on our preliminary, unpublished data obtained under similar experimental settings.

### 2.5. Hepatocytes Isolation, Culture and Treatment

Nile tilapia hepatocyte isolation and culture were performed as described previously [21]. Livers were sliced (0.5 mm) using a McIlwain Tissue Chopper (Mickle Laboratory Engineering Co., Ltd., Surrey, UK) and digested with collagenase IV (0.5 mg/mL, Sigma, Cream Ridge, NJ, USA) and DNase II (20 U/mL, Worthington, Columbus, OH, USA) for 20 min at 28 °C with gentle shaking. The dispersed cells were filtered by different sizes of nylon gauze and purified by centrifugation at different speed (70× *g*, 50× *g* and 30× *g*, respectively). The cells were seeded into 24-well culture plates (8 × 10^5^ cells/well) in L15 medium (added 1% penicillin-streptomycin and 10% fetal bovine serum) at a 28 °C incubator. Cold shock treatments were performed by incubating the cells in a biochemical incubator at 12 ± 1 °C for 24 h. The tested compounds (MedChemExpress, Monmouth Junction, NJ, USA) were as follows: cortisol (2 μM), L-Epinephrine Bitartrate (Epi, 1 μM), MG-132 (2 μM) and BAM15 (10 μM). Cells were harvested at 6 h and 24 h.

### 2.6. Quantitative Real-Time PCR

Gene expression was analyzed by qPCR using 10 μL of reaction mix containing 5 μL of SYBR Green mix (Dong Sheng Biotech, Guangzhou, China), 0.3 μL of each primer, 1 μL of cDNA, and 3.4 μL of water. RT-PCR assays of target and β-actin (reference) genes were performed using the Roche Light Cycler 480 (Roche, Rasel, Switzerland). Relative mRNA expressions were estimated using the 2^−ΔΔCt^ approach. The oligonucleotide primer sequences utilized in this study are provided in Table 1.

### 2.7. siRNA Electroporation in Nile Tilapia Hepatocytes

Double-stranded siRNA directed against Nile tilapia UCP1 mRNA (sense strand: 5′-GCAGAGACAGCUGUGCUUUTT-3′, anti-sense strand: 5′-AAAGCACAGCUGUCUCUGCTT-3′) were obtained from GenePharma Corporation (Shanghai, China). As a control, the scrambled siRNA was provided by the GenePharma Corporation. The siRNA was used at a final concentration of 2.25 μM. Transient transfection of Nile tilapia hepatocytes with UCP1 siRNA, or control siRNA was performed using the Neon Transfection System (Invitrogen, USA). Briefly, the freshly isolated hepatocytes were counted and transferred into sterile EP tubes, centrifuged at 200 g for 5 min, and then resuspended with R-buffer at a concentration of 8 × $10^{6}$ cells/mL (for seeding into a 24-well plate). 5 μL of UCP1 siRNA or control siRNA was mixed with 100 μL of cell resuspension. The mixture was aspirated into the Gold-Tip of the MicroPorator Pipette for Neon Transfection. Following microinjection at 1200 V, 40 ms, 1 pulse, the 105 µL contents were promptly dispensed into a pre-filled 24-well plate with L15 medium with 10% FBS. Transfected cells were cultured and treated with 1 μM Epi for 16 h post-transfection.

### 2.8. Western Blot

Nile tilapia hepatocytes were lysed by RIPA lysis buffer (Beyotime Biotechnology, Shanghai, China) in the presence of 1% protease inhibitors (Biotool, Shanghai, China), and the protein level was quantified by a commercial bicinchoninic acid (BCA) assay kit (Beyotime Biotechnology, Shanghai, China). Proteins were separated by SDS-PAGE and transferred to PVDF membranes (Merck Millipore, Burlington, MA, USA). Membranes were blocked for 1 h with 5% non-fat milk in TBST. The following primary antibodies were used: UCP1 (Sigma, USA) and GAPDH (Cell Signaling Technology, Danvers, MA, USA). After incubating in 4 °C overnight with primary antibodies, the PVDF membranes were washed three times with TBST and incubated with the secondary antibodies. Blots were then incubated using the ECL immunoblotting detection system (Tanon, Shanghai, China). The band intensities were measured using ImageJ software (ImageJ 1.54d) for quantification.

### 2.9. Mitochondrial Membrane Potential (MMP)

The JC-1 Mitochondrial Membrane Potential Assay kit (Beyotime Biotechnology, Shanghai, China) was employed to assess. Nile tilapia hepatocytes MMP. In short, hepatocytes were plated (8 × $10^{5}$ cells/mL) in 24 wells, and after treatments, the detection of MMP was carried out according to the manufacturer’s protocol. Cells were photographed in red and green channels using a confocal microscope at a 63× oil immersion lens (Leica TSC SP8, Wetzlar, Germany).

### 2.10. ATP Assay

According to the kit instructions (Beyotime Biotechnology, Shanghai, China), cells or 20 mg tissue samples were collected in EP tubes, adding the appropriate amount of ATP lysate, blowing with a pipette gun or breaking with a tissue crusher to make the cells fully lysed, centrifuging the cells at 4 °C and 12,000× *g* for 5 min after lysis, and taking the supernatant for the subsequent ATP determination. RLU or CPM were measured by a microplate reader (Perklin Elmer Vivtor X5, Waltham, MA, USA) with a chemiluminescence meter (luminometer). Calculate the sample ATP concentration using the standard curve. To mitigate errors arising from variations in protein quantity during sample preparation, the ATP concentration can be normalized to nmol/mg of protein.

### 2.11. BAM15 Injection and Fed Experiment

Juvenile male Nile tilapia (Oreochromis niloticus, body weight: 10 ± 2 g) were used for the experiments. For the injection experiment, fishes were intraperitoneally (i.p.) injected with BAM15 (GLPBIO, Montclair, CA, USA) at doses of 0.2 mg/kg or 1 mg/kg body weight, administered 1 h before the cooling treatment. Control (vehicle) fishes received an injection of the same solvent used for BAM15 preparation, following the procedure described by Kenwood [22]. For the feeding experiment, fishes were fed a commercial diet (Feng Hua, Ningbo, China) either unsupplemented (control) or supplemented with BAM15 at 0.05% (*w*/*w*) for 7 consecutive days before acute cold exposure. The dietary supplementation level was determined based on doses reported in mice [23]. Following exposure to acute cold stress, samples were collected at 6 h and 24 h post-treatment.

### 2.12. Physiological Stress Indicator Analysis

Using 1 mL sterile syringes, blood was drawn from the tilapia and transferred into 1.5 mL sterile EP tubes. The samples were allowed to stand at room temperature for 2 h, followed by centrifugation at 3000× *g* for 15 min. The serum was carefully collected. Serum glucose levels were assayed using a commercial kit employing the glucose oxidase reaction (Beijing Boxbio Science & Technology Co., Ltd., Beijing, China), while serum lactate and triglycerides concentrations were quantified using a commercial lactic acid assay kit (Nanjing Jiancheng Bioengineering Institute, Nanjing, China).

### 2.13. H&E Staining

Intestinal samples were fixed in 4% paraformaldehyde, embedded in paraffin, and sectioned at 8 μm thickness. H&E staining was performed following the protocol of [24]. Slides were visualized under a light microscope (Nikon Eclipse Ni-E, Tokyo, Japan).

### 2.14. Statistical Analysis

All reported values are presented as the mean with the standard error of the mean (SEM) and were compared statistically using either the Student’s *t*-test or one-way ANOVA, followed by Duncan’s multiple range test for further analysis. All analyses were conducted using the SPSS Statistics 23 (IBM, Armonk, NY, USA).

## 3. Results

### 3.1. UCP1 Was Involved in Cold Stress Response in Nile Tilapia

**Figure 1:**
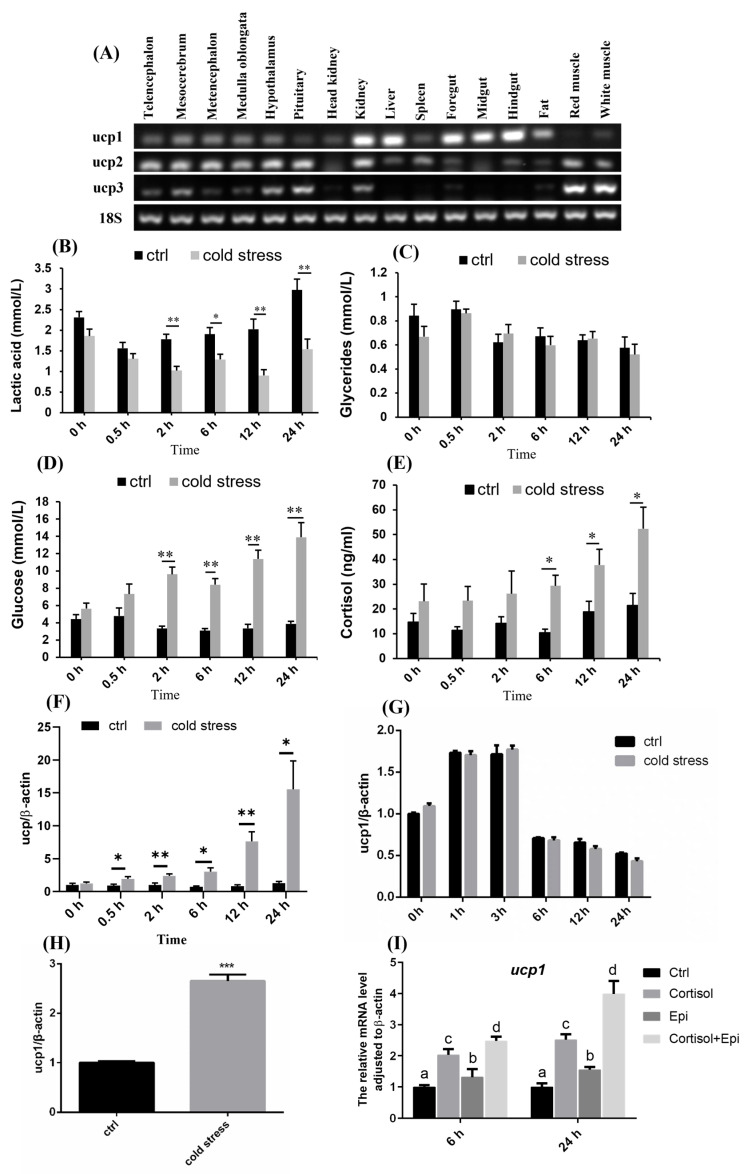
**UCP1 was involved in cold stress response in Nile tilapia**. (**A**): Distribution of *ucp1* in tissues. (**B**–**E**): Concentration of lactic acid, triglycerides, glucose, and cortisol in serum under acute cold stress (n = 8). (**F**): UCP1 mRNA in liver during acute cold stress (n = 4–6). (**G**): UCP1 mRNA level in hepatocytes suffered from 13 °C (cold stress) (n = 4). (**H**): Effect of serum incubation on hepatocytes UCP1 expression. The serum was obtained from Nile tilapia cultured at 28 °C (ctrl) and 13 °C (cold stress), and the concentration of serum was 10%, n = 5–6. (**I**) Effect of L-Epinephrine Bitartrate (Epi) and/or cortisol treatment on UCP1. All data were presented as means ± S.E.M. For (**A**–**H**), statistical significances were determined by Student’s *t*-test. * *p* < 0.05, ** *p* < 0.01, *** *p* < 0.001. For (**I**), statistical significances were determined by one-way analysis of variance followed by Duncan’s multiple range test, different letters stand for *p* < 0.05.

The distribution characteristic of Nile tilapia *ucp1* was confirmed by semi-quantitative PCR. Comparable to other teleost, ucp1 gene exhibits extensive expression across both central and peripheral tissues (Figure 1A), such as kidney, liver and gut, and with the lowest mRNA level in pituitary, red muscle, and white muscle (Figure 1A).

As UCP1 plays a thermogenic role in mammals, acute cold stimulation models were built *in vivo* and *in vitro* to determine whether UCP1 still serves the same function in Nile tilapia. Acute cold exposure dramatically reduced the amount of lactic acid in serum within 2 h and continued for 24 h (Figure 1B). While glucose and cortisol, the crucial mediators of stress response [25], were markedly elevated while maintaining an up-regulated trend (Figure 1D,E), indicating the body was suffering from cold stress and the hormones may have affected metabolism. The triglyceride levels remained insignificantly altered (Figure 1C). At the same time, we observed that UCP1 mRNA in the liver, a crucial organ for metabolic adaptations to stresses and a primary target for cortisol, increased at 0.5 h after cold stimulation (Figure 1F), and remained higher than the control group for the following 24 h. However, hepatocytes *ucp1* did not exhibit variations in a biochemical incubator at 13 °C compared to 28 °C (Figure 1G). Interestingly, it was significantly increased (Figure 1H) when incubated with cold-stressed Nile tilapia serum. These findings indicate that a non-cell autonomous process likely involves circulating substances to cause cold-induced UCP1 expression. As shown in Figure 1I, UCP1 mRNA was up-regulated at 6 h and 24 h treated with Epi and/or cortisol incubation, an adrenergic receptor agonist, confirming the effect of stress hormones in UCP1 expression. Furthermore, Epi and cortisol had additive effects when combined during incubation for 24 h.

### 3.2. UCP1 Acted on Cold Stress Response via Mitochondria Membrane Potential and Proteasome

**Figure 2:**
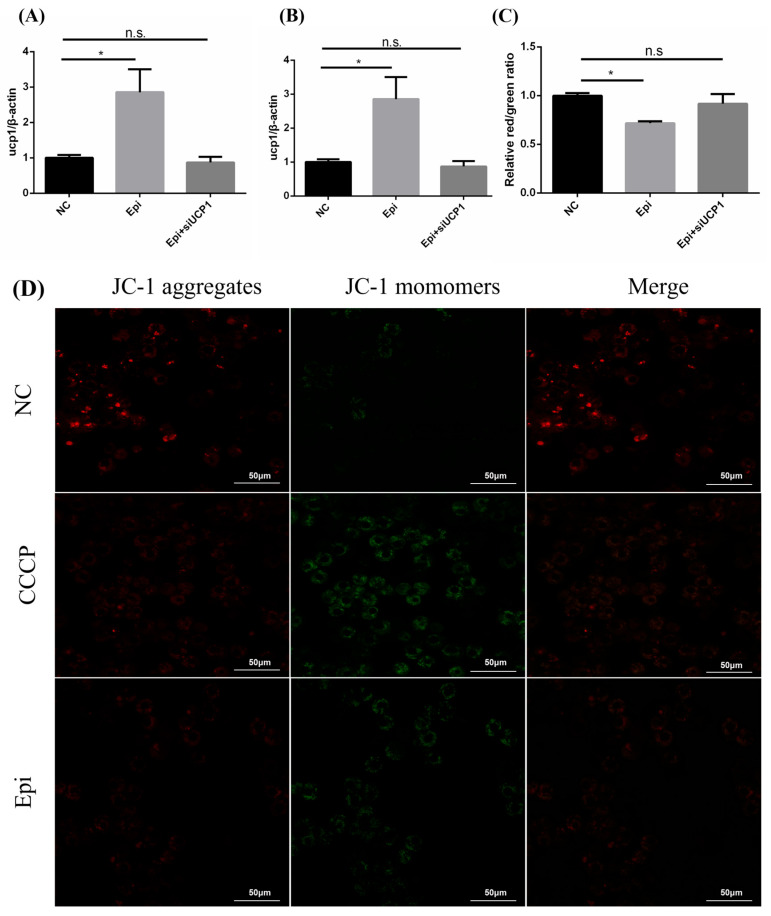

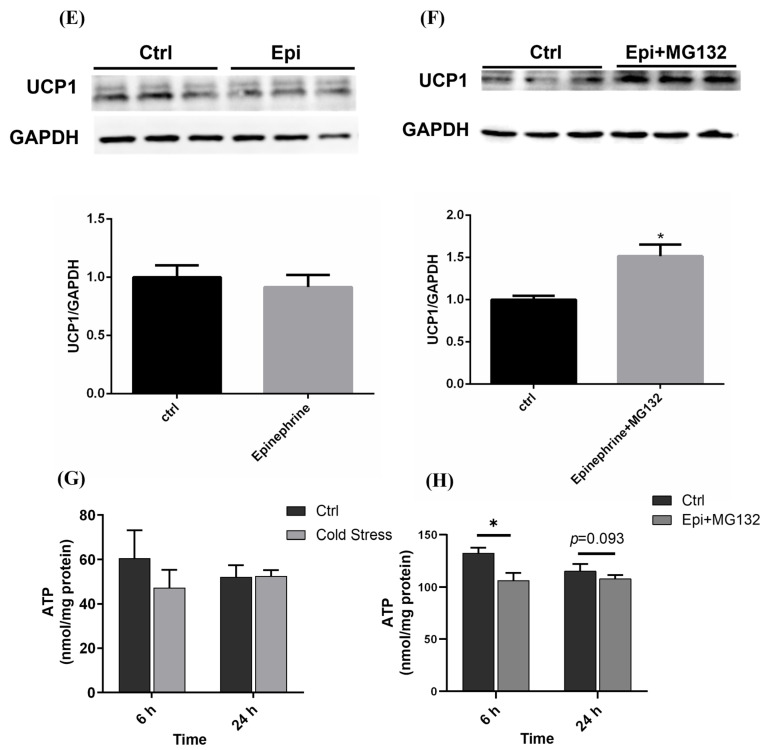
**UCP1 is a functional and conservative uncoupling protein in Nile tilapia**. (**A**,**D**) The mitochondrial membrane potential of hepatocytes treated with Epi (2 μM) for 24 h, CCCP (Carbonyl cyanide 3-chlorophenylhydrazone) served as a positive control in the assay to validate membrane depolarization, scale bar = 50 μm. (**B**,**C**) The expression of *ucp1* and MMP in tilapia hepatocytes transfected with siRNA, and then exposed to Epi for 24 h. (n = 3–4). (**E**) Western blotting for UCP1 from hepatocytes treated with Epi (2 μM) for 24 h. (**F**) Western blotting for UCP1 in hepatocytes incubated with Epi (2 μM) and MG-132 (2 μM) for 24 h. (**G**,**H**) The concentration of ATP in the liver and hepatocytes, after acute cold exposure or co-treatment with Epi and MG132, respectively. Date was presented as means ± S.E.M. For (**A**–**C**), statistical significance was determined by ANOVA. For (**G**,**H**), statistical significances were determined by Student’s *t*-test. * Indicates different levels of significance. n.s., not significant.

The Epi-induced upregulation of UCP1 in tilapia primary hepatocytes provides a model to investigate whether UCP1 mediates proton leak by examining MMP. It was found that MMP was inhibited by Epi treatment (Figure 2A,D). UCP1 siRNA was transfected by electroporation and significantly decreased UCP1 mRNA level (Figure 2B), and reversed mitochondrial potential compared to Epi treatment (Figure 2C). The above results demonstrate that UCP1 enhances mitochondrial proton leak in Nile tilapia hepatocytes. Despite an increase in UCP1 mRNA, protein level remained unaffected (Figure 2E). As seen in Figure 2F, protease inhibition during Epi treatment increased UCP1 protein levels, indicating the degradation of UCP1 mediated by the cytoplasmic proteasome.

Furthermore, in both the hepatocytes (Figure 2H) and the acute cold stimulation paradigm (Figure 2G), the ATP content decreased to varying degrees and then recovered to approximately the same level as the control group after 24 h. The signs all point to UCP1 as a conservative and functional uncoupling protein in the Nile tilapia.

### 3.3. UCP1 Participated in the Regulation of Glucose and Lipid Metabolism Under Cold Stress

**Figure 3:**
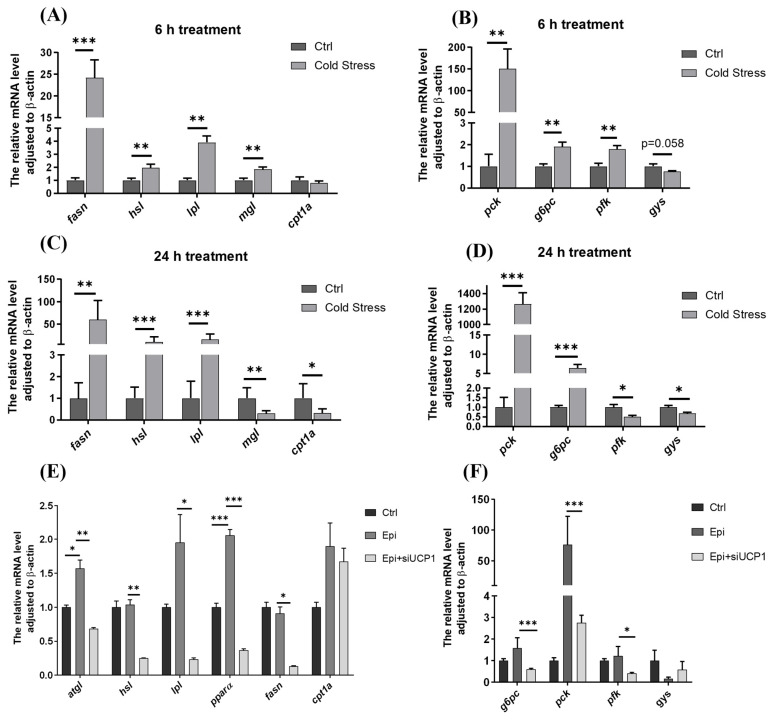

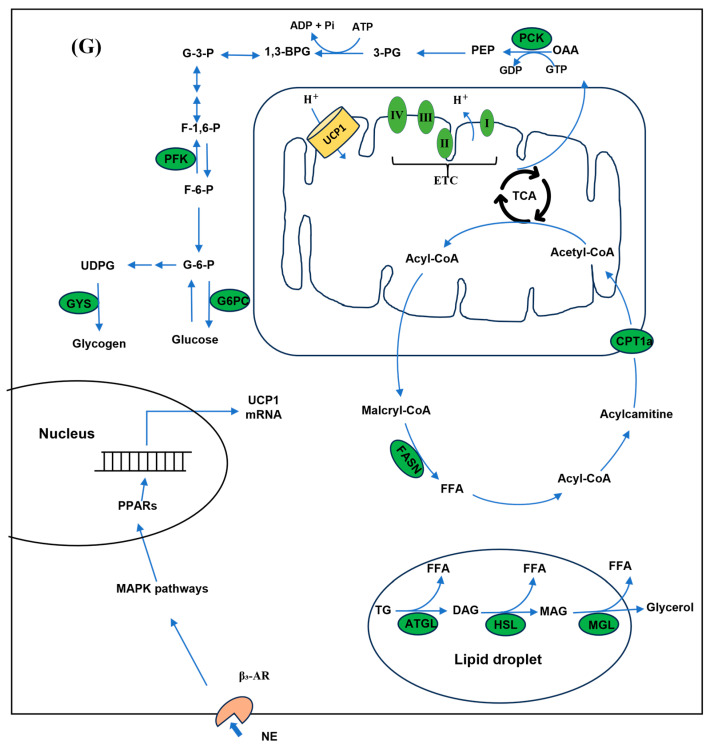
**UCP1 participated in the regulation of glucose and lipid metabolism in Nile tilapia under cold stress**. (**A**,**B**) Genes related to lipid and glucose metabolism under 6 h. (**C**,**D**) Genes related to lipid and glucose metabolism under 24 h (n = 8–12). (**E**,**F**) Genes involved in glucose and lipid metabolism in cold-stimulated cell models after siUCP1 (n = 4–6). (**G**) Association between mitochondrial uncoupling and glucose/lipid metabolism. *Fasn* (fatty acid synthase), *hsl* (hormone-sensitive lipase), *lpl* (lipoprotein lipase), *mgl* (monoacylglycerol lipase), *cpt1a* (carnitine o-palmitoyl transferase 1), *pck* (phosphoenolpyruvate carboxy kinase), *g6pc* (glucose-6-phosphatase), *pfk* (phosphofructokinase-1), *gys* (glycogen synthase 1), *atgl* (adipose triglyceride lipase), ETC (electron transfer chain), TCA (tricarboxylic acid cycle), FFA (free fatty acid), TG (triglyceride), DAG (diacylglycerol), MAG (monoglyceride), OOA (Oxaloacetic acid), Arrows denote the progression between steps in the pathway. All data are expressed as mean ± S.E.M, one-way ANOVA, * *p* < 0.05, ** *p* < 0.01, *** *p* < 0.001.

Regulating lipid metabolism is a vital way to remission response under cold stress in fish [26,27,28]; however, little is known about the upstream regulatory pathways of cold-induced lipid metabolism changes at present. Notably, during the 6 h cold exposure period, we detected significant upregulation of key genes regulating both lipid catabolism and anabolism (Figure 3A), gluconeogenesis and glycolysis (Figure 3B). These cortisol-mediated metabolic reprogramming effects on carbohydrate and lipid metabolism were sustained throughout the cold exposure paradigm (Figure 3C,D). To assess the potential UCP1 dependency of these metabolic shifts, we employed siRNA-mediated UCP1 knockdown in primary hepatocyte cultures. It was found that the expression of lipolysis-related enzymes, such as *atgl*, *lpl*, *pparα*, were UCP1-dependent decreased after siUCP1 (Figure 3E). However, not all enzyme expression changes (e.g., *hsl*, *fasn*) were directly correlated with UCP1 mRNA levels, indicating that more complex regulatory mechanisms may be involved. We observed UCP1-dependent regulation of key metabolic enzymes, with gluconeogenic enzymes (*pck*, *g6pc*) showing significant expression changes while glycogen synthase expression progressively declined (Figure 3F). UCP1 potentially regulates glucose and lipid metabolism via the metabolic pathway network (Figure 3G).

### 3.4. Mitochondrial Uncoupling Mediated by BAM15 Enhanced Acute Cold Stress Tolerance

**Figure 4:**
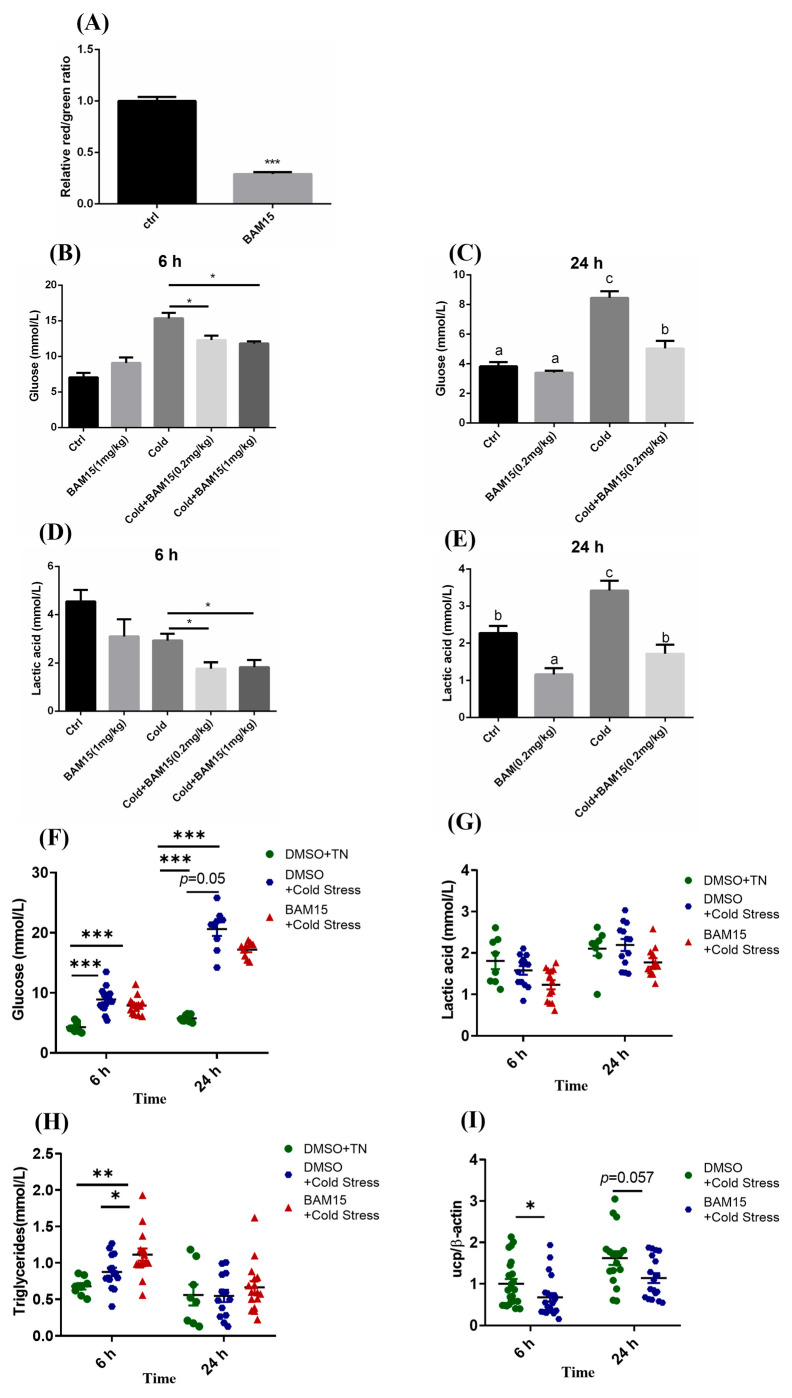

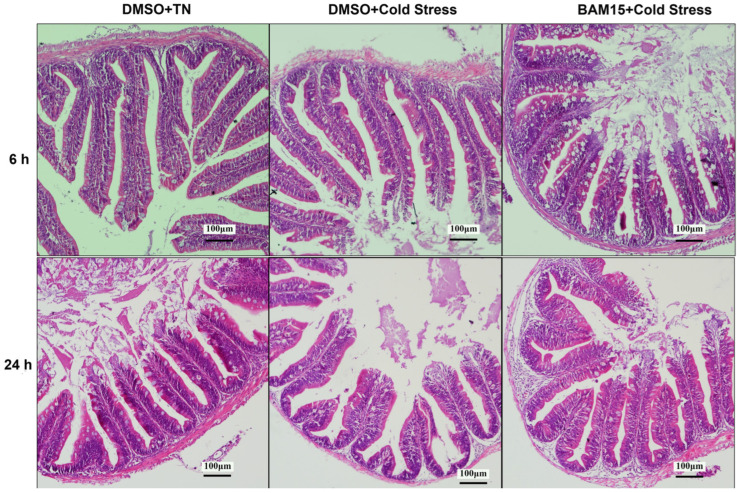
**BAM15-mediated mitochondrial uncoupling increased acute cold stress tolerance**. (**A**) BAM15 treatment reduced mitochondrial membrane potential of primary hepatocytes. Hepatocytes were treated with BAM15 for 4 h at the concentration of 10 μM. (**B**,**C**) are the serum glucose levels after 6 and 24 h of cold stimulation, respectively. (**D**,**E**) are the serum lactic acid levels after 6 and 24 h of cold stimulation. BAM15 was injected intraperitoneally 1 h before cold stimulation (n = 4–8). (**F**–**H**) are the levels of serum glucose and lactic acid, triglycerides in tilapia under cold stress after BAM15 feeding. (**I**) The expression of UCP1 mRNA in tilapia liver. (**J**) H&E staining of the foregut. Scale bar = 100 μm. TN: thermoneutrality (28 °C), n = 8–13. Data are expressed as mean ± standard error and analyzed by one-way ANOVA and Duncan’s multiple range test, * *p* < 0.05, ** *p* < 0.01, *** *p* < 0.001.

BAM15, a known and currently considered the safest mitochondrial uncoupler in mice, enhances oxygen consumption and tissue energy expenditure [22,23,29]. As previously mentioned, UCP1-mediated changes in glucose and lipid metabolism contribute to cold resistance in tilapia. This study employs BAM15 to investigate whether enhanced mitochondrial uncoupling could improve cold tolerance in tilapia. The incubation of BAM15 reduced the mitochondrial membrane potential of hepatocytes (Figure 4A), indicating that BAM15 is an effective mitochondrial uncoupler in Nile tilapia. The group injected with BAM15 showed lower levels of glucose (Figure 4B,C), lactic acid (Figure 4D,E) than cold stress group. Those results demonstrate that BAM15 could protect the fish from acute cold stress through intraperitoneal injection.

With the purpose of applying in economic fishery production, trace amounts of BAM15 were added to the commercial diet. Short-period BAM15 feeding before acute cold stress appreciably lessened the content of serum glucose compared to the commercial diet (Figure 4G), while the TG of serum decidedly rose in 6 h but not 24 h (Figure 4H). Similar as to intraperitoneal injection, lactic acid in serum had a declined tendency in BAM15-fed group (Figure 4I). In the meantime, the endogenous UCP1 mRNA level decreased in the liver after being fed the special diet added BAM15 under acute cold stress (Figure 4F). H&E staining results demonstrated that the foregut (Figure 4J), midgut and hindgut (Supplementary Figure S1) of tilapia fed with BAM15-supplemented diet exhibited a clear and intact structure without pathological alterations, indicating the safety of BAM15. All the signals above indicated that over response induced by acute cold stress was remissible in ways of intraperitoneal injection and peroral uncoupler agent BAM15 undeniably.

## 4. Discussion

In mammals, the functions of UCP proteins have been demonstrated in cellular processes ranging from thermogenesis to metabolism and antioxidant defense [30,31], but the physiological functions of UCP in fish is not yet confirmed. Previous investigations have demonstrated that various physiological states affect fish *ucp1*, such as fasting and cold stress. However, the degree of modulation exhibits considerable variability across UCP families in tissues and species [32]. In this study, we found that the Nile tilapia UCP1 was mainly expressed in the liver, kidney and the intestinal tract, which was similar to other fish [14,16], while the liver, kidney and intestine are all sensitive to a wide range of stressors [33,34,35]. Taking all the results mentioned above into consideration, we speculate that the biological role of UCP1 in Nile tilapia is pressure resistance and homeostasis maintenance, which has been emphasized by Santos et al. [32].

There has been much research exploring the expression of UCP1 under cold conditions in fish. We have noticed that most of the research was performed under chronic cold stress, or low temperature acclimation. And in most of the research, UCP1 was down-regulated [15,18]. In our study, a model of acute cold stimulation was established, in which UCP1 was upregulated in the liver, and this upregulation lasted for at least 24 h. Acute and chronic stress may have variant effects on fish endocrine and immune responses and metabolism [36], which might explain the expression pattern of UCP1 in different cold conditions.

In mammals, the expression of UCP1 is governed by a complex interplay between the sympathetic nervous system and hormones [37]. Reports indicate that increased activities of sympathetic nerves upregulate UCP1 mRNA expression in response to cold exposure [38], as well as β-adrenergic and thyroid-hormone stimulation [39]. While norepinephrine, epinephrine and cortisol level were also found elevated in cold-treated tilapia [40]. We also observed cortisol level increasing in our cold stress model. According to the different phenomena between straightforward cold stimulation and stress hormone treatment experiment in hepatocytes, we speculate that the expression of UCP1 is not directly regulated by cold stimulation but regulated by stress hormones. The different responses between low-temperature treatment and stress hormone treatment also suggest that more attention should be paid to the role of hormones when establishing cold stress models *in vitro*.

The mRNA level of UCP1 varies dynamically in response to environmental stress [17], but the protein level of UCP1 was rarely detected in fish. It has been documented that the half-life of UCP1 is about 3 h [41], and the half-life of UCP2 is 30 min in rat thymocytes [42]. The 26S proteasome is potentially involved in the degradation of short-lived proteins and is required for the degradation of UCP1 and UCP2 [41,43]. According to our research, UCP1 mRNA was increased after Epi treatment, but the protein level was not changed unless MG-132, the proteasome inhibitor, was used to block protein degradation. Based on these results, we hypothesize that, similar to mammals, the Nile tilapia UCP1 protein has a short half-life and is also degradated by proteasomes.

In mammals exposed to a cold environment, sympathetic nerves and adrenergic receptors on adipose tissue are activated. This activation triggers the AMPK signaling pathway, which promotes UCP1 expression [44] and lipolysis, leading to the production of free fatty acids [45]. The proton conductance activity of UCP1 depends on the presence of free fatty acids and can be inhibited by purine nucleotides (e.g., ATP, ADP, GTP, and GDP) [46]. However, elevated levels of free fatty acids can counteract this inhibition [47]. Lipolysis and lipid synthesis are essential for fish to maintain energy homeostasis and physiological functions. Therefore, the high expression of lipolysis and adipogenesis in tilapia under cold stress may reflect a compensatory mechanism to meet increased energy demands. Our study confirmed activated lipolysis and elevated UCP1 expression, suggesting that Nile tilapia UCP1 may be regulated by fatty acids, similar to observations in *Common carp* [48]. In contrast, the expression of lipolysis-related enzymes and fatty acid synthase decreased after UCP1 knockdown. This reduction may be attributed to the decreased availability of fatty acids required for UCP1 activation, as well as impaired fatty acid utilization and accumulation. Reduced uncoupling restores intracellular energy levels and decreases AMPK-activated lipolysis [49]. This is consistent with studies showing that abnormal lipolysis activation causes excessive fat loss and higher mortality [50]. Additionally, acute cold stress in tilapia elevates blood glucose by increasing gluconeogenesis and reducing glycogen synthesis. These physiological mechanisms lead to glucose accumulation in the liver, which is subsequently released into the circulation and utilized by other tissues. In hepatocytes, UCP1 knockdown diminishes the uncoupling effect, resulting in sufficient ATP production to meet energy demands. Consequently, gluconeogenesis and glycolysis are substantially reduced, restoring the metabolic rate to a normal state.

The rapid alterations in serum levels of catecholamines and corticosteroid stress hormones accompany the primary stress response in fish, which leads to energy mobilization, glycogen stores reduction, and glycemia augmentation [51]. It has been shown that low temperature reduces the oxygen transfer efficiency of fish, which results in the accumulation of lactic acid, an aerobic metabolism product in fish [52]. Therefore, glucose and lactate can frequently serve as indicators of stress in fish [40,53], which were significantly higher in our acute cold model. A newly discovered mitochondrial protonophore uncoupler, BAM15, possesses the capability to safeguard mammals from cold-induced microtubule damage [54]. In the present study, BAM15 was verified as an efficient uncoupling agent to affect MMR in teleost hepatocytes for the first time. BAM15 injection and feed could inhibit the upregulation of glucose and lactic acid levels to a certain extent, which proves that BAM15 can alleviate the stress response of Nile tilapia to low temperature. Uncoupler was demonstrated as a new strategy to enhance cold resistance in fish.

Efficient and high-throughput transfection of nucleic acids into fish primary cells would be a valuable experimental tool for functional research. Viral vectors and lipid reagents have been employed for siRNA delivery in mammal cells, while these approaches exhibit limited transfection efficiency and high cytotoxicity in fish primary cells. The Neon™ Transfection System from Invitrogen is a cutting-edge next-generation electroporation method that has garnered significant attention for transfecting human primary cells [55,56]. No reports have demonstrated the application of Neon™ Transfection System on fish primary cells. Here, we found that the Neon™ Transfection System could efficiently transmit UCP1 siRNA to the Nile tilapia hepatocytes and alleviate the decrease in mitochondria membrane potential caused by UCP1. Electroporation stands as an effective method for siRNA delivery, albeit with potential drawbacks such as cell damage and ion imbalances [57,58]; however, optimization of the parameters for fish cells requires further investigation.

## 5. Conclusions

In conclusion, this study demonstrates that UCP1 is a functional and conserved protein in Nile tilapia. It contributes to resistance against acute cold stress by promoting mitochondrial uncoupling and regulating lipid and glucose metabolism. These findings enrich understanding of UCP1 function in teleost. However, regulatory factors upstream and downstream of UCP1 still need to be further confirmed in the future. These findings provide new insights into improving cold tolerance in Nile tilapia, which may contribute to reducing winter mortality and enhancing sustainability in aquaculture.

## Figures and Tables

**Table 1:** **Primer sequences used for PCR**.

Name	Accession	Forward Primer (5′-3′)	Reverse Primer (5′-3′)
*ucp1-orf*	XM_005456938	ATGGTAGGACTTAAACCCTCAGA	TCAGTTAACCACTTCGATCCTCT
*ucp1*	XM_005456938	GTAGGACTTAAACCCTCAGACA	CCAGAGGGAATGTGACCATA
*atgl*	XM_005475579.4	GGAGAATGTGCTCGTGTC	ATGCTGGTGTTGGTGAAA
*fasn*	XM_003454056.5	CCAGACTTCAGAGACTCCATTC	TGCGTGAACTGTGTCTTCAA
*hsl*	XM_031726103.1	GCCCTATCTAAAGAGTTGGTCC	CGATGTCGTCACACATAAGTTG
*mgl*	KX845691.1	GCGTATGGAGAGGGAGAT	ATGGTGAAGAGCGTGGTA
*pparα*	NM_001290066.1	GGCTGCTATTATCTGCTGTG	TGTCATCTGGGTGGTTGG
*cpt1a*	XM_003440552.5	TTTCCAGGCCTCCTTACCCA	TTGTACTGCTCATTGTCCAGCAGA
*pck*	XM_003448375.3	CCAGACCGCCGACAAATTATC	GTTGGTGATGCCCAGGATCA
*g6pc*	XM_013273429.2	CGCCAGCATGAAGAAGTATTTC	CACCACTTCTGGGCTTTCTC
*gys*	ENSONIT00000002127	TGACAAGGAGGCTGGTGAGAGG	ACTCGTGCATGGCTGAGAACTT
*pfk*	XM_003447353.4	TGGCTAAGAAGCATTATAACTTGAA	TGGTCATTATGGCGTCAATTATC
18S	XR_003218766.1	AGAAACGGCTACCACATCC	CACCAGACTTGCCCTCCA
*β-actin*	XM_003443127	ACCTTCTACAACGAGCTGAGAG	GCCTGGATGGCAACGTACA

## Data Availability

The data that support the findings of this study are available from the corresponding author upon reasonable request.

## Supplementary Materials

The following supporting information can be downloaded at: https://www.mdpi.com/article/10.3390/metabo15100668/s1, Supplementary Figure S1. Midgut and Hindgut of tilapia.

## Abbreviations

The following abbreviations are used in this manuscript.

Abbreviation	Definition
ATP	adenosine triphosphate
FoxO	Forkhead box o
H&E	Hematoxylin and Eosin staining
MAPK	mitogen-activated protein kinase
ORF	open reading frame
qPCR	quantitative real-time PCR
ROS	reactive oxygen species
P/S	Penicillin-Streptomycin
TBST	Tris-buffered saline with 0.05% Tween20
SDS-PAGE	sodium dodecyl sulphate
GDP	Guanosine diphosphate
