# Supplementary figures and images for: Uncoupling Protein 1 Promotes Nile Tilapia Resistance to Acute Cold Stress by Regulating Liver Metabolism
Source: Metabolites. 2025 Oct 13;15(10):668. doi: 10.3390/metabo15100668 (PMC12566292; doi:10.3390/metabo15100668)

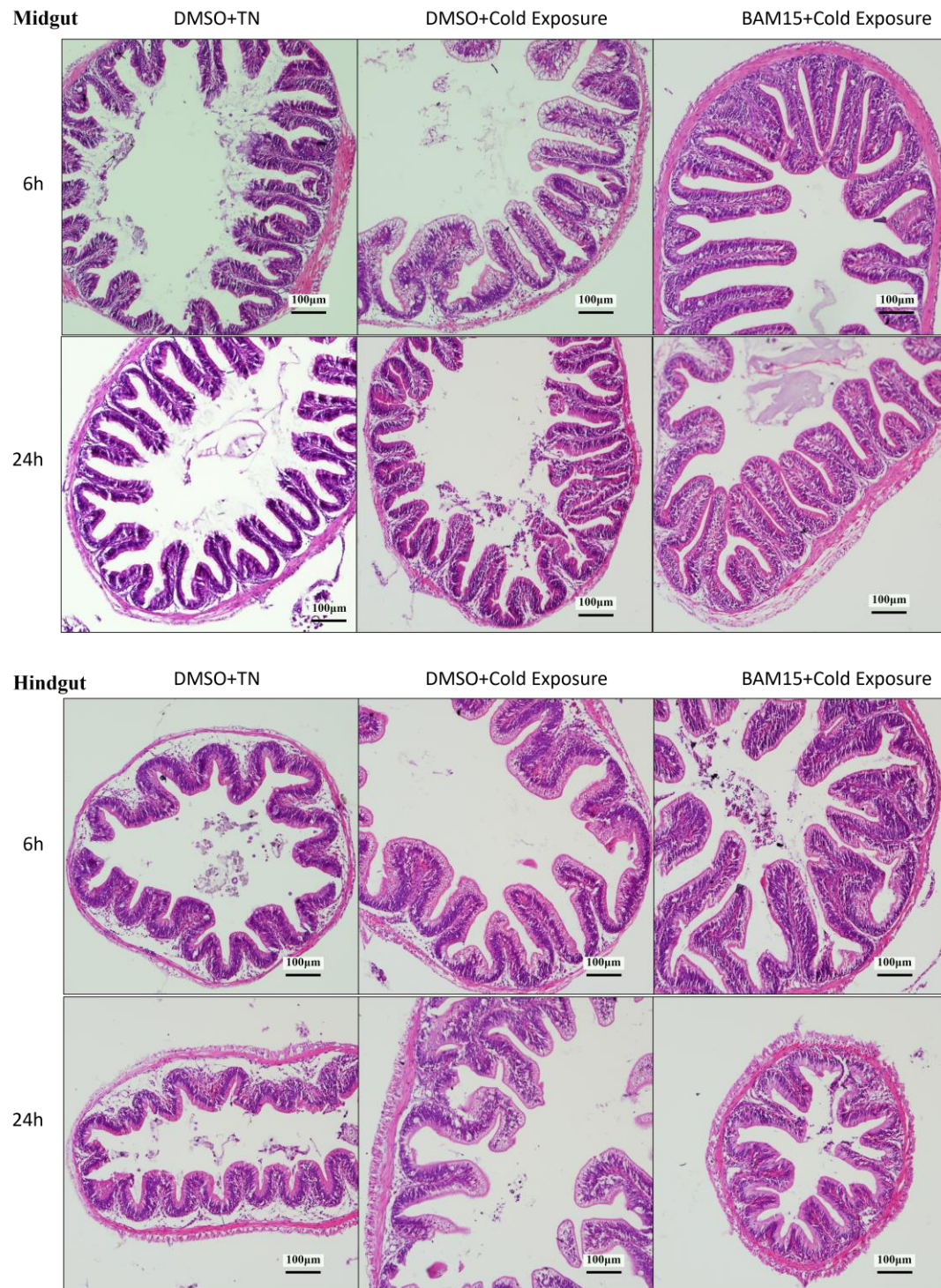

**Figure S1.** Midgut and Hindgut of tilapia.

Supplement: Supplementary file 1 [file metabolites-15-00668-s001.zip › metabolites-3876837-supplementary.pdf]
